# ExtremeDB: A Unified Web Repository of Extremophilic Archaea and Bacteria

**DOI:** 10.1371/journal.pone.0063083

**Published:** 2013-05-16

**Authors:** Manash Chandra Majhi, Abhisek Kumar Behera, Niha Mohan Kulshreshtha, Dr. Mahmooduzafar, Rita Kumar, Anil Kumar

**Affiliations:** 1 Environmental Biotechnology Division, Institute of Genomics and Integrative Biology, (IGIB – CSIR), Delhi, India; 2 Department of Botany, Jamia Hamdard University, Delhi, India; 3 Department of Biotechnology, University of Pune, Ganeshkhind, Pune, India; 4 Patent Division, National Institute of Immunology (NII), New Delhi, India; Louisiana State University and A & M College, United States of America

## Abstract

Extremophiles are the microorganisms which can survive under extreme conditions of temperature, pressure, pH, salinity etc. They have gained much attention for their potential role in biotechnological and industrial applications. The large amount of experimental data in the literature is so diverse, that it becomes difficult and time consuming for the researcher to implement it in various areas of research. Therefore, a systematic arrangement of data and redirection in a similar fashion through web interface can assist researchers in analyzing the data as per their requirement. ExtremeDB is a freely available web based relational database which integrates general characteristics, genome-proteome information, industrial applications and recent scientific investigations of the seven major groups of 865 extremophillic microorganisms. The search options are user friendly and analyses tools such as Compare and Extreme BLAST have been incorporated for comparative analysis of two or more extremophiles and determining the sequence similarity of a given protein/nucleotide in relation to other extremophiles respectively. The effort put forth herein in the form of database, would open up new avenues on the potential utility of extremophiles in applied research. ExtremeDB is freely accessible via http://extrem.igib.res.in.

## Introduction

Extremophiles are peculiar microorganisms that flourish under harshest conditions of temperature, pressure, salinity, pH etc. [1]. The majority of extremophiles are members of the archaeal domain, but certain species from bacterial domain too have the potential to adapt to extreme conditions [2], [3]. The unusual physical and chemical properties of extremophiles that enable them to sustain unfavorable conditions make them potentially useful in industry, technology and medicine [3]–[5]. The biocatalysts from extremophiles (extremozymes) are exceptionally stable under extreme conditions and therefore offer new opportunities for biotransformation and biodegradation [5], [6]. For example cellulases, amylases, xylanases, proteases, pectinases, keratinases, lipases, esterases, catalases, peroxidases and phytases have found wide potential for application in various biotechnological processes. Enzymes from cryophiles or psychrophiles can be used to enhance the cleaning power of laundry detergents in cold water industry. Similarly, thermophilic enzymes have also found many applications in industrial processes (Table S1). Once the chemistry and structure of these enzymes is understood, they can often be made synthetically or can be further modified for use in manufacturing and other fields. Extremophiles are also attractive subjects of research in astrobiological as well as evolutionary studies as they repair or resist damage from extreme heat, radiation, toxic substances and thus provide a glimpse of what the primitive forms of life looked like [7], [8]. Deciphering the genomes of extremophiles may reveal unique genes that such organisms have evolved. Such scientific investigations are of great significance to several domains of research such as biology, microbiology, biotechnology and biochemistry and require access to useful repository of information, followed by its maintenance for further use. A quick search in PubMed with the keyword “extremophiles” shows as many as 1119 hits. However, the task of extracting information from literature resources is tedious and time consuming. One is often required to browse different databases for extracting different type of information e.g. NCBI Genome [9], GOLD [10] for genomic information, KEGG [11] for pathway information, NCBI protein for protein sequence, RCSB-PDB [12] for structural information and BRENDA [13] for enzyme information. Despite the critical value of extremophiles in industrial applications and healthcare, no effort has been made to compile their unique properties on a single platform.

To facilitate the retrieval of information from primary literature resources and its integration with most of the available web resources on a convenient platform, we developed ExtremeDB. ExtremeDB is a web accessible, user friendly navigation tool which currently houses 865 extremophilic species. The compilation of information at a single place will not only aid researchers in hypothesis generation but will also be equally useful to industry people who wish to exploit extremophiles for novel applications.

## Materials and Methods

### Database Architecture and Web Interface

**Figure 1:**
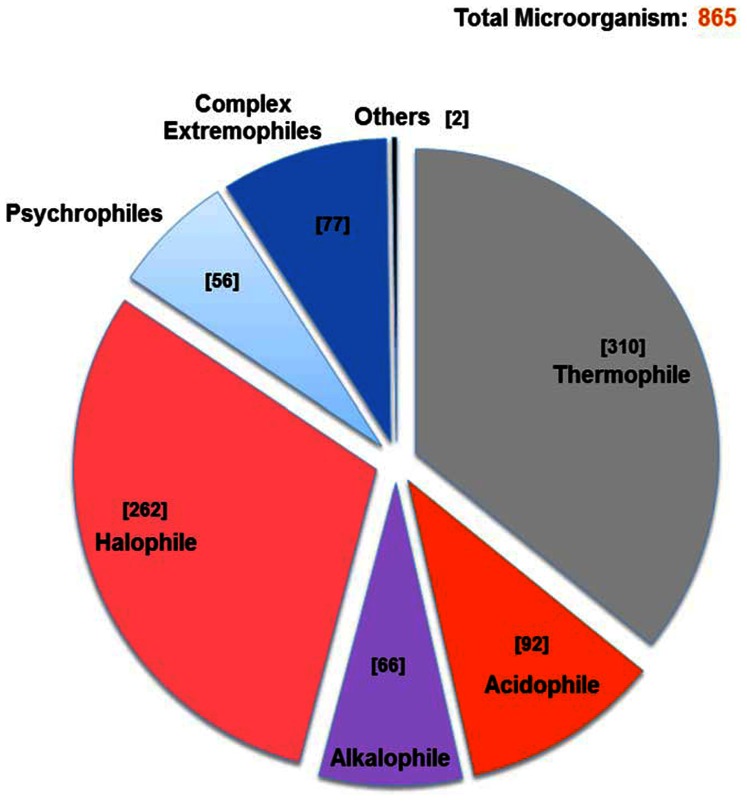
Categorical distribution of available microorganisms in ExtremeDB.

**Figure 2:**
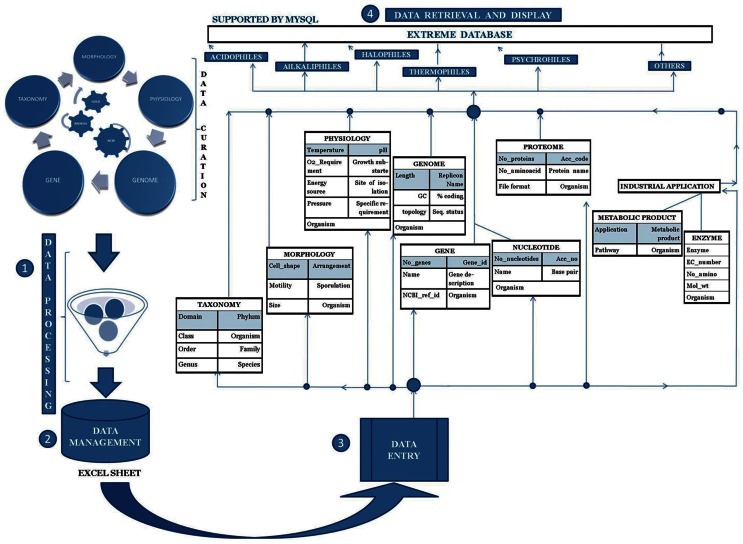
Flow chart describing the methodology and process in ExtremeDB.

The database consists of 865 extremophilic microorganisms, which are either from bacterial or archaeal domain. These have been categorized into seven categories- thermophiles, acidophiles, alkalophiles, halophiles, psychrophiles, complex extremophiles and others. The data was derived from public databases and different literature resources and then analyzed. Currently the database has information about 310 thermophiles, 92 acidophiles, 66 alkalophiles, 262 halophiles, 56 psychrophiles, 77 in complex extremophiles and 2 microorganisms in the others category. Figure 1 shows the categorical distribution of available microorganisms in ExtremeDB. A well-organized schema was designed to disseminate substantial information. The database has been developed using AMP (Apache-MySQL-PHP) module along with AJAX, XML, and JavaScript. The back end data have been stored in a relational database called MySQL (http://www.mysql.com). Server scripting was done in PHP: Hypertext Preprocessor, Version 5.0 (http://www.php.net). The client side scripting was done in JavaScript and HTML (Hyper Text Mark-up Language, Version 4.0). Apache, Version 2.2.2 (http://www.apache.org) has been used as a web server to parse the query. The database is hosted on 64 bit windows 2003 Server. A flow chart depicting the methodology and the process used in construction of ExtremeDB is illustrated in Figure 2.

### Database Content

**Figure 3:**
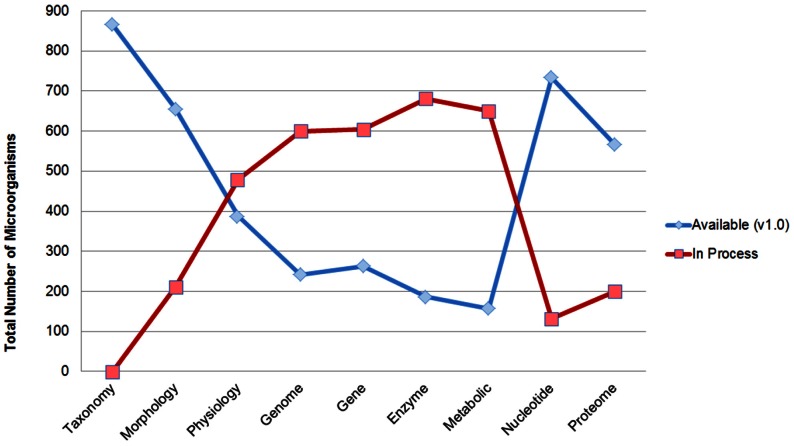
Characteristics of available and In-process Microorganisms (865) in ExtremeDB. Blue line corresponds to the information available in the current version (v1.0), whereas the red line indicates the ongoing work which will be amended in the next build.

Every species in the database are allotted a unique code describing its attributes. For example, *Thermoproteus tenax* has been accorded with accession code “acido05”. It represents that the species belongs to an acidophile in the hierarchy number five. Each entry constitutes of information like Taxonomy, Morphology, Physiological characteristics, Nucleotide, Gene, Genome, Proteome, Enzymes and Metabolic Products. Figure 3 shows the availability of information in the current version of ExtremeDB. Following information is included in Taxonomy - Domain, Phylum, Class, Order, Family, Genus, Species and Strain. The Tax_id and PMID fields are linked to NCBI Taxonomy Browser and PubMed respectively. The morphology section provides information like - Cell shape, Arrangement, Motility, Sporulation and Size. Important information like Site of Isolation, Temperature, pH, O_2_ Requirement, Growth Substrate, Energy source, Salinity, Pressure, and Specific Requirement etc. have been incorporated in Physiological characters. The Genome field of any organism displays the current sequencing status of its genome. The Nucleotide section represents the total number of available nucleotides along with accession number and name. It also allows the user to choose different display formats. The Gene header includes information on available genes of an organism. As in the case with Nucleotides, the link field under this header displays different display formats which are individually hyperlinked to the NCBI gene database. The information about the available proteins is included in the Proteome header. It also provides a different file format as in case of nucleotide. The Enzyme section furnishes enzyme information available so far which includes E.C. Number, Enzyme Name, No. of amino acids, Molecular weight and Sources. The Metabolic Products produced by the organisms are also listed in the Metabolic Product section. The last section is for literature which shows an important citation for the selected species.

## Results and Discussion

### Operation of Database

ExtremeDB is configured using relational database management system which is a collection of data items organized as a set of formally-described tables from which data can be accessed or reassembled in many different ways. The system includes a web interface that allows user to consult the database and display the results in an interactive way. Various options i.e. ORG.LIST, BLAST and COMPARE at the top of the interface provide a convenient way to start searching and browsing the database. The client-server architecture allows the system to run in a central server and be used simultaneously by any number of client machines. The database has three main cores i.e List of organisms, Search and Analysis tools. These three cores together make the database more efficient to support extremophilic research. The users can add new information by filling simple web forms immediately after registration. Registration is completely free; once registered, user can suggest changes/data updation if required, however they will be incorporated only after verification by the team members.

### Organism List (ORG.LIST)

An organism list in ExtremeDB is denoted as “ORG.LIST” where the information has been categorized into seven major categories- Acidophiles, Alkalophiles, Halophiles, Psychrophiles, Thermophiles, Complex Extremophiles and Others. The last category represented as “OTHERS” includes species originating from various habitats such as endolith, hypolith, lithoautotroph, metalotolerant, oligotroph, osmophile, piezophile, polyextremophile, xerophile. The organism list shows the name of the organisms, their code, category and kingdom where each organism is hyperlinked to its detailed information. Organisms can be listed either alphabetically or on the basis of their category or their domain. Once the organism of interest is clicked, subsequent relevant information of that organism including its Taxonomy, Morphology, Physiological Characteristics, Nucleotide, Gene, Genome, Proteome, Enzymes and Metabolic Products would be displayed. Any additional information, if required, can be fetched through the hyperlinks provided in specific fields.

### Compare

**Figure 4:**
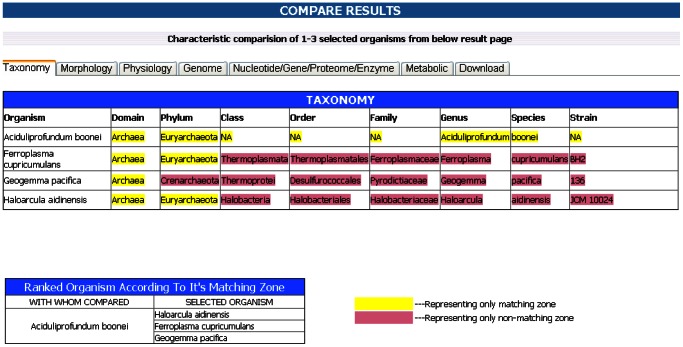
A carved figure showing the result of compare tool in ExtremeDB.

ExtremeDB provides tools for comparative analysis of two or more organisms based on the user's inputs. This tool comes handy during primary data analysis and research. Handling compare module is very simple where users can compare two or more (maximum up to four) different species on the basis of their different attributes irrespective of their origins. Selection boxes provide suggestions generated from the database to avoid textual errors. The user can add or remove properties from mandate boxes and can compare the data (Figure 4). The query species are compared against the master species and output is displayed in a tabular form. In the first table, attributes matching with master species are highlighted in yellow color and the mismatch is depicted in the red color. Second table ranks query species according to their similarity with master species. Species showing maximum similarity appear first in the table followed by less similar species. We can download the output of comparing results according to user inputs.

### Extreme BLAST

Extreme BLAST is a tool which is based on the minimum expected score of similarity between the target and query sequences. The algorithm implemented, is a mirror to Basic Local Alignment Search Tool (BLAST, 2.2.23). ExtremeBLAST has a comprehensive collection of non-redundant protein, nucleotide sequence for 865 microorganisms from all the seven categories. Extreme BLAST allows choosing different databases (ExtremeDB, ExtremeDB-Archaea, ExtremeDB-Bacteria, and NCBI) from pull down menu. The first three databases are specific for extremophilic microorganisms and the last database is provided to compare against all the sequences present in NCBI. Five different programs are available in this tool such as BLASTP, BLASTN, BLASTX, TBLASTX, and TBLASTN. To build such a tool, protein and nucleotide information on extremophiles was mined, processed and then formatted in the desired format. The user can submit or upload a sequence in FASTA format. Full BLAST results including the alignment can be saved as a text file. The filters in Extreme BLAST are meant for filtering low complexity regions and other parameters are set to default.

### Advanced Search

**Figure 5:**
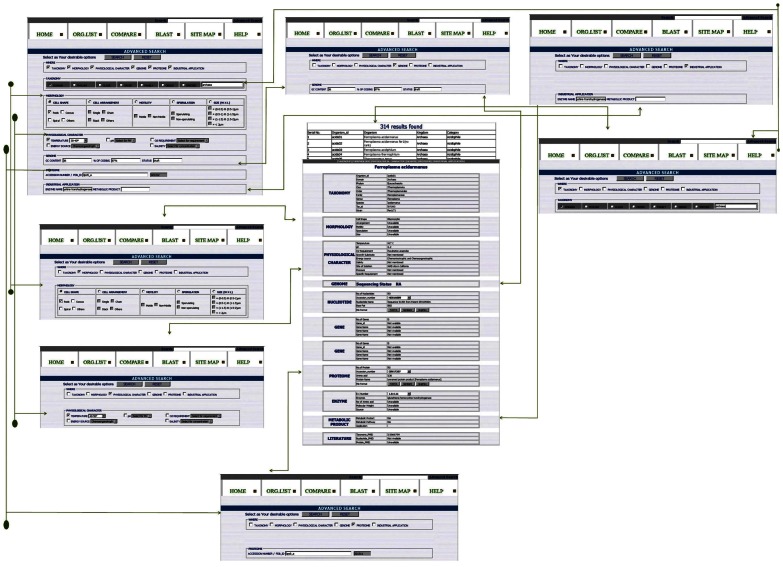
A carved figure showing the features of advanced search in ExtremeDB.

Searching in an advanced way is important to narrow down onto specific result with less or no redundancy or duplicity. The advanced search in ExtremeDB correlates various mandates to ascertain specific information if available. Searching enables retrieving data through different behaviors of each mandate. E.g. In taxonomy fields, microorganisms can be searched according to their classification like class, order, etc. Or, if one is interested in the organisms of a particular niche, having a particular physiological property, then the same can be carried out easily in advanced search. Features of advanced search in ExtremeDB are shown in Figure 5.

### Submission and Update of ExtremeDB

Currently the database gets updated manually by reviewing the scientific publication available and via user's contribution. The online data submission tool is available at http://extrem.igib.res.in/administrator.html, where the user has to register by filling a simple web form. This tool will allow a user to submit a newly identified or un-reported extremophilic organism for ExtremeDB database. However, to maintain the quality, these data are analyzed and checked before incorporating into the database. Information related nucleotide and proteins gets updated time to time using programs written in Perl. Meanwhile, to make regular updates our team is also searching for new entries of extremophiles from published literature.

## Conclusion

We have developed a database dedicated to extremophilic microorganisms which aids in the systematic representation of the large amount of experimental data from the primary literature and various web resources. ExtremeDB will facilitate data analysis, hypothesis generation and would open up new avenues on the potential utility of extremophiles in basic and applied research.

### Availability and Requirements

ExtremeDB is freely available at http://extrem.igib.res.in.

## Supporting Information

Table S1 Important Extremozymes.
(DOC)
