# Correction: ExtremeDB: A Unified Web Repository of Extremophilic Archaea and Bacteria

**DOI:** 10.1371/journal.pone.0179119

**Published:** 2017-06-01

**Authors:** Manash Chandra Majhi, Abhisek Kumar Behera, Niha Mohan Kulshreshtha, Dr. Mahmooduzafar, Rita Kumar, Anil Kumar

The phrase “ExtremeDB” appears incorrectly in the title and throughout the article. PLOS has received notice from McObject LLC that “ExtremeDB” is a registered trademark of McObject LLC. The correct phrase in each instance in the paper should instead be “ExtremophileDB”, and the correct database link is http://extremophiledb.igib.res.in/.
