# Supplementary material for: ExtremeDB: A Unified Web Repository of Extremophilic Archaea and Bacteria
Source: PLoS One. 2013 May 16;8(5):e63083. doi: 10.1371/journal.pone.0063083 (PMC3656046; doi:10.1371/journal.pone.0063083)
Supplement: Table S1 — Important Extremozymes. (DOC) [file pone.0063083.s001.doc]

**Table S1. Important Extremozymes**

| Sl. No | Extremozymes | Species | Extremophile | Biotechnological And Industrial Uses | Reference(s) |
| --- | --- | --- | --- | --- | --- |
| 1 | Cellulases | Trichoderma maritima MSB8 | Thermophile | In biodetergents | [1] |
|  |  | Thermotoga neapolitana | Thermophile | In formulation of detergents for cold washing reducing the energy consumption and, wear and tear of textile fibers | [2] |
|  |  | Caldocellum saccharolyticum | Thermophile | Biopolishing and stone washing of textile products | [3] |
|  |  | Anaerocellum thermophilum | Extreme Thermophile | In alcohol production to improve juice yields and effective colour extractions of juices | [4] |
|  |  |  |  | Pretreatment of cellulosic biomass and forage crops improving the nutritional quality and digestibility |  |
| 2 | α-Amylases | Pyrococcus woesei | Hyperthermophile | For the hydrolysis and modification of starch to produce glucose and various other products | [5] |
|  |  | Pyrococcus furiosus | Hyperthermophile | In biodetergents | [6, 7] |
|  |  | Thermococcus profundus | Hyperthermophile | In formulation of detergents for cold washing reducing the energy consumption and, wear and tear of textile fibers | [8] |
|  |  | Desulfurococcus mucosus | Hyperthermophile | Application in detergents to assist in stain removal | [9] |
|  |  | Pyrococcus sp. KOD1 | Hyperthermophile | Enzymatic conversation of Starch | [10] |
|  |  | Staphylothermus marinus | Hyperthermophile | Cultivation of these microorganisms were done for the production of alpha amylase which has many significant role in biotechnology and industrial process. These enzymes are thermostable. | [9] |
|  |  | Sulfolobus solfataricus | Hyperthermophile | Production of alpha- amylases which can be used several industrial process | [11] |
|  |  | Thermococcus celer | Hyperthermophile | Enzyme used as a starch degrading/ liquefaction agent | [8, 9] |
|  |  | Thermococcus profundus | Hyperthermophile | Hydrolysis of glycosidic bonds | [12] |
|  |  | Thermococcus aggregans | Hyperthermophile | Enzyme used as a starch degrading/ liquefaction agent | [9] |
|  |  | Halobacillus sp. strain MA-2 | Moderately Halophile | hydrolyzing starch under high-stress conditions ; | [13] |
|  |  |  |  | useful for treating saline water or waste solutions containing starch residues | [67] |
|  |  |  |  | Production of extracelluar amylases | [14] |
| 3 | Xylanases | Dictyoglomus thermophilum Rt46B.1 | Thermophile | Biobleaching of pulp and paper, thus lowering the environmental pollution by halogens | [15, 16] |
|  |  | Thermotoga sp. strain FjSS3-B.1 | Thermophile | Biobleaching of pulp and paper, thus lowering the environmental pollution by halogens | [17] |
|  |  | Thermotoga neapolitana | Thermophile | Biobleaching of pulp and paper, thus lowering the environmental pollution by halogens | [2] |
|  |  | Thermotoga thermarum | Thermophile | Biobleaching of pulp and paper, thus lowering the environmental pollution by halogens | [16] |
| 4 | β-Xylanase | Halorhabdus utahensis | Extreme halophile | Cellulose degradation | [18] |
| 5 | β-Xylosidase | Halorhabdus utahensis | Extreme halophile | Cellulose degradation | [18] |
| 6 | Protease CPI | Pseudoalteromonas sp. strain CP76 | Moderate Halophile | Production of proteolytic enzymes that has wide application in industries | [19] |
| 7 | Serine Protease | Desulfurococcus strain Tok12S1 | Hyperthermophile | In biodetergents | [20] |
|  |  | Desulfurococcus strain SY | Hyperthermophile | fat hydrolysis, esterification, interesterification,trans-esterification and organic biosynthesis | [21] |
|  |  | Staphylothermus marinus | Hyperthermophile | Removal of pitch from pulp produced in the paper industry | [22] |
|  |  | Pyrobaculum aerophilum | Hyperthermophile | Hydrolysis of milk fat in the dairy industry | [23] |
|  |  | Fervidobacterium pennavorans | Extreme Thermophile | Tenderization of meat | [24] |
| 8 | Carboxy Protease | Sulfolobus solfataricus | Hyperthermophile | Removal of non-cellulosic impurities from raw cotton | [25] |
| 9 | Acidic Protease | Sulfolobus acidocaldarius | Hyperthermophile | Removal of subcutaneous fat in the leather industry | [26, 27] |
| 10 | Thiol Protease | Pyrococcus sp. KOD1 | Hyperthermophile | In formulation of detergents for cold washing reducing the energy consumption and, wear and tear of textile fibers | [28, 29] |
| 11 | Chymotrypsinogen B-like protease | Natronomonas pharaonis | Haloalkaliphile | As a detergent additive | [30] |
| 12 | Esterases | Pyrococcus furiosus | Hyperthermophile | In physicochemical methods for the bioremediation of solids and waste waters pollutedby hydrocarbons, oils and lipids | [31] |
|  |  | Caldocellum saccharolyticum | Extreme Thermophile | Production of esterases which has industrial application in the degradation of industrial pollutants like cereal waste, plastic and other toxic materials | [32] |
|  |  | Sulfolobus acidocaldarius | Extreme Thermophile | Production of esterases which has industrial application in the degradation of industrial pollutants like cereal waste, plastic and other toxic materials | [33] |
| 13 | Glucoamylase | Clostridium thermohydrosulfuricum 39E | Thermoanaerobic | For the production of glucose syrups | [34] |
|  |  | Clostridium thermosaccharolyticum | Thermoanaerobic | Complete conversion of polysaccharides to glucose | [35] |
|  |  | Thermoanaerobacterium thermosaccharolyticum DSM 571 | Thermophile | Pullulanse enzymes produced is used in clevasge of alpha-1,6-glucosidic bonds | [36] |
| 14 | Chitinases | B. licheniformis X-7u | Thermophile | Chitin degradation | [37] |
|  |  | Bacillus sp. BG-11 | Thermophile | Chitin degradation | [38] |
|  |  | Streptomyces thermoviolaceus OPC-520 | Thermophile | Chitin degradation | [39] |
| 15 | DNA polymerases I | Thermus aquaticus | Thermophile | Thermostable DNA polymerases | [40, 41] |
| 16 | Keratinase | Fervidobacterium pennavorans | Thermophile | Recycling of keratin-wastes | [24] |
| 17 | Pullulanase type II | Pyrococcus woesei | Hyperthermophile | Hydrolyze both α-1,4- and α-1,6-glycosidic bonds in branched polymers | [42] |
|  |  | Pyrococcus furiosus | Hyperthermophile | Hydrolyze both α-1,4- and α-1,6-glycosidic bonds in branched polymers | [42] |
| 18 | Pullulanases | Thermus caldophilus GK-24 | Thermophile | Improve the industrial starch hydrolysis process | [43] |
|  |  | Fervidobacterium pennavorans Ven5 | Thermophile | Improve the industrial starch hydrolysis process | [43] |
| 19 | α-Glucosidases | Pyrococcus woesei | Hyperthermophile | In the final step of glycogen degradation | [44] |
|  |  | Pyrococcus furiosus | Hyperthermophile | In the final step of glycogen degradation | [45] |
|  |  | Sulfolobus solfataricus strain 98/2 | Hyperthermophile | final step of glycogen degradation; | [46] |
|  |  |  |  | Hydrolyzing terminal glucose residues |  |
| 20 | Trehalose | Sulfolobus shibatae | Thermoacidophile | used as a stabilizing agent in numerous industries | [47] |
| 21 | Alcohol dehydrogenases | Thermococcus stetteri | Thermophile | Catalyze the oxidation of alcohols to ketones and reverse | [48] |
|  |  | Sulfolobus solfataricus | Hyperthermophile | Catalyze the oxidation of alcohols to ketones and reverse | [49] |
| 22 | Hydrogenases | Methanococcus jannaschii | Piezophile | Catalyze the oxidation of alcohols to ketones and reverse | [50] |
| 23 | Carboxylesterase | Pyrobaculum calidifontis | Hyperthermophile | excellent model systems for studying structure function relationships as well as in vitro and in vivo evolution and possible biotechnological applications | [51] |
| 24 | Endoglucanase | Alicyclobacillus acidocaldarius | Thermoacidophile | have potential effectiveness as additives to laundry detergents | [52] |
| 25 | Lipase | Psychrobacter okhotskensis | Psychrophile | formulation of detergents for cold | [53] |
|  |  |  |  | washing |  |
| 26 | Azoreductase | Bacillus sp. Strain SF | Alkali-Thermophile | responsible for the decolorization activity | [54] |
| 27 | Catalase-peroxidase | Bacillus sp. Strain SF | Alkali-Thermophile | textile finishing effluent | [55] |
| 28 | β-galactosidase | Arthrobacter sp. C2-2 | Psychrophile | hydrolysis of lactose in milk | [56] |
| 29 | Oxidase | Pedobacter cryoconitis | Psychrophile | bioremediation of solids and waste waters polluted by hydrocarbons, oils and lipids | [57] |
| 30 | β-Mannanase | Rhodothermus marinus | Thermophile | bioconversion of lignocellulose material | [58] |
| 31 | Carboxylesterase | Pyrobaculum calidifontis | Hyperthermophile | synthesis and hydrolysis of stereospecific compounds; | [51] |
|  |  |  |  | metabolic processing of drugs and antimicrobial agents |  |
| 32 | L-Aminoacylase | Thermococcus litoralis | Hyperthermophile | Used in commercial biotransformation | [59] |
| 33 | Polygalacturonase | Cystofilobasidium larimarini | Psychrophile | Cheese ripening, fruit juice and wine industry | [60] |
|  |  | Cystofilobasidium capitatum | Psychrophile | Cheese ripening, fruit juice and wine industry | [60] |
| 34 | Catalase | Thermus brockianus | Thermophile | degrades hydrogen peroxide to oxygen and water | [61] |
| 35 | Pectate lyase | Bacillus alcalophillus | Alkaliphilic | fruit juice industry, textile and paper treatment | [62] |
| 36 | Pectate lyase | Pseudoalteromonas haloplanktis ANT/505 | Psychrophile | Cheese ripening, fruit juice and wine industry | [63] |
| 37 | Alkaline phosphatase | Vibrio sp. G15-21 | Psychrophile | Molecular biology | [64] |
| 38 | 3-Isopropylmalate dehydrogenase | Vibrio sp. I5 | Psychrophile | Asymmetric chemical synthesis | [65] |
| 39 | Chitinase A | Arthrobacter sp.TAD20 | Psychrophile | Food, health products | [66] |

**SUPPLEMENTARY REFERENCES**

1. Bronnenmeier, K., Kern,A., Liebl, W., and Staudenbauer, W.L. (1995) Purification of Thermotoga maritima enzymes for the degradation of cellulose materials. Appl Environ Microbiol., **61**, 1399-1407.
2. Bok, J.D., Yernool, D. A., and Eveleigh, D. E. (1998) Purification, characterization and molecular analysis of thermostable cellulases Cel A and Cel B from Thermotoga neapolitana. Appl Environ Microbiol., **64**, 4774-4781.
3. Teo, V.S., Saul, D.J. and Bergquist, P.L. (1995) CelA, another gene coding for a multidomain cellulase from the extreme thermophile Caldocellum saccharolyticum. Appl Microbiol Biotechnol., **43**, 291-296.
4. Zverlov, V., Mahr,S., Riedel, R. and Bronnenmeier, K. (1998) Properties and gene structure of a bifunctional cellulolytic enzyme (CelA) from the extreme thermophile Anaerocellum thermophilum with separate glycosyl hydrolase family 9 and 48 catalytic domains. Microbiology, **144**, 457-465.
5. Koch, R., Spreinat, K., Lemke, K. and Antranikian, G. (1991) Purification and properties of a hyperthermoactive a-amylase from the archaeobaterium Pyrococcus woesei. Arch Microbiol., **155**, 572-578.
6. Laderman, K.A., Davis, B.R., Krutzsch, H.C., Lewis, M.S., Griko, Y.V., Privalov, P.L. and Anfinsen, C.B. (1993) The purification and characterization of an extremely thermostable aamylase from the hyperthermophilic archaebaterium Pyrococcus furiosus. J BiolChem., **268**, 24394-24401.
7. Jorgensen, S., Vorgiasg, Constantin., E. and Antranikian, G. (1997) Cloning, sequencing and expression of an extracellular a-amylase from the hyperthermophilic archeon Pyrococcus furiosus in Escherichia coli and Bacillus subtilis. J Biol Chem., **272**, 16335-16342.
8. Chung, Y. C., Kobayashi, T., Kanai,H., Akiba, T., AND Kudo,T. (1995) Purification and properties of extracellular amylase from the hyperthermophilic archeon Thermococcus profundus DT5432. Appl Environ Microbiol., **61**, 1502-1506.
9. Canganella,F., Andrade, C.M. and Antranikian, G. (1994) Characterization of amylolytic and pullulytic enzymes from thermophilic archaea and from a new Fervidobacterium species. Appl Environ Microbiol., **42**, 239-245.
10. Tachibana,Y.,Fujiwarab,S.,Takagib,M. and Imanaka,T. (1996) Cloning and expression of the α--amylase gene from the hyperthermophilic archeon Pyrococcus sp. KOD1 and characterization of the enzyme. J Ferment Bioeng., **82**, 224-232.
11. Haseltine,C., Rolfsmeier,M. and Blum,P. (1996) The glucose effect and regulation of aamylase synthesis in hyperthermophilic archaeon Sulfolobus solfataricus. J Bacteriol., **178**, 945-950.
12. Kwaka,Y.S., Akibab,T. and Kudob,T. (1998) Purification and characterization from hyperthermophilic archaeon Thermococcus profundus, which hydrolyses both a-1,4 and a-1,6 glucosidic linkages. J Ferment Bioeng., **86**, 363-367.
13. Amoozegar, M.A., Malekzadeh, F. and Malik, K.A. (2003) Production of amylase by newly isolated moderate halophile, Halobacillus sp. Strain MA-2., J. Microbiol. Methods., **52**, 353-359.
14. Prakash, B.,Vidyasagar,M.,Madhukumar,M.S.,Muralikrishna, G.,Sreeramulu, K.(2008) Production, purification, and characterization of two extremely halotolerant, thermostable, and alkali-stable α-amylases from Chromohalobacter sp. TVSP 101. Process Biochem., **44(2)**, 210-215.
15. Gibbs, M. D., Reeves, R.A., and Bergquist, P.L. (1995) Cloning, sequencing and expression of a xylanase gene from the extreme thermophile Dictyoglomus thermophilum Rt46B.1 and activity of the enzyme on fiber-bound substrate. Appl Environ Microbiol., **61**, 4403-4408.
16. Sunna, A. and Antranikian, G. (1997) Growth and production of xylanolytic enzymes by the extreme thermophilic anaerobic bacterium Thermotoga thermarum. Appl Microbiol Biotechnol., **45**, 671-676.
17. Simpson, H. D., Haufler, U. R. and R M Daniel (1991) An extremely thermostable xylanase from the thermophilic eubacterium Thermotoga. Biochem J., **277**, 177-185.
18. Waino, M. and Ingvorsen,K. (2003) Production of β-xylanase and β-xylosidase by the extremely halophilic archaeon Halorhabdus utahensis. Extremophiles, **7**, 87-93.
19. Porro, C. S., Mellado,E., Bertoldo,C., Antranikian, G., and Ventosa,A. (2003) Screening and characterization of the protease CP1 produced by the moderately halophilic bacterium Pseudoalteromonas sp. strain CP76. Extremophiles, 7, 221–228.
20. Cowan, D.A., Smolenski, K.A., Daniel, R.M. and Morgan, H.W.(1987) An extremely thermostable extracellular proteinase from a strain of the archaebacterium Desulfurococcus growing at 88°C. Biochem J., **247**, 121-133.
21. Hanzawa, S., Hoaki, T., Jannasch, H.W., and Maruyama, T. (1996) An extremely thermostable serine protease from a hyperthermophilic archaeon Desulfurococcus strain SY, isolated from a deep-sea hydrothermal vent. J Mar Biotechnol., **4**, 121-126.
22. Mayr, J., Lupas, A., Kellermann, J., Eckerskorn, C., Baumeister, W. and Jurgen, P. (1996) A hyperthermostable protease of the subtilisin family bound to the surface of the layer of the archaeon Staphylothermus marinus. Curr Biol., **6**, 739-749.
23. Volkl, P., Markiewicz, P., Stetter, K. and Miller, J.H. (1994) The sequence of a subtilisintype protease (aerolysin) from the hyperthermophilic archaeon Pyrobaculum aerophilum reveals sites important to thermostability. Protein Sci., **3**, 1329-1340.
24. Friedrich, A. B. and Antranikian, G. (1996) Keratin degradation by Fervidobacterium pennavorans, a novel thermophilic anaerobic species of the order Thermotogales. Appl Environ Microbiol., **62**, 2875-2882.
25. Burlinia, N., Magnania, P.,Villaa, A., Macchia, F., Tortora, P. and Guerritorea, A. (1992) A heat-stable serine proteinase from the extreme thermophilic archaebacterium Sulfolobus solfataricus. Biochim Biophys Acta., **1122**, 283-292.
26. Fusek, M., Lin, X. L. and Tang, J. (1990) Enzymic properties of thermopsin. J Biol Chem., **265**, 1496-1501.
27. Lin, X.L. and Tang, J. (1990) Purification, characterization and gene cloning of thermopsine, a thermostable acidic protease from Sulfolobus acidocaldarius. J Biol Chem., **265**,1490-1495.
28. Fujiwara, S., Okuyama, S., and Imanaka, T. (1996) The world of archaea: genome analysis, evolution and thermostable enzymes. Gene., **179**, 165-170.
29. Morikawa, M., Izawa, Y., Rashid, N., Hoaki, T. and Imanaka, T. (1994) Purification and characterization of a thermostable thiol protease from a newly isolated hyperthermophilic Pyrococcus sp. Appl Environ Microbiol., **60**, 4559-4566.
30. Stan-Lotter, H., Doppler, E., Jarosch, M., Radax, C., Gruber, C. and Inatomi,K. (1999) Isolation of a chymotrypsinogen B-like enzyme from the archaeon Natronomonas pharaonis and other halobacteria. Extremophiles, **3**, 153–61.
31. Ikeda, M., Clark, D.S. (1998) Molecular cloning of extremely thermostable esterase gene from hyperthermophilic archaeon Pyrococcus furiosus in Escherichia coli. Biotechnol Bioeng., **57**, 624–9.
32. Luthi, E., Jasmat, N. B. and Bergquist, P. L. (1990) Overproduction of an acetylxylan esterase from from the extreme thermophilic bacterium Caldocellum saccharolyticum. Appl Microbiol Biotechnol., **19**, 2677-2683.
33. Sobek, H. and Gorisch, H. (1988) Purification and characterization of a heat stable esterase from the themoacidophilic archaeobacterium Sulfolobus acidocaldarius. Appl Environ Microbiol., **61**, 729-733.
34. Hyun, H. H. and Zeikus, J. G. (1985) Regulation and genetic enhancement of glucoamylase and pullulanase production in Clostridium thermohydrosulfuricum. J Bacteriol., **164**, 1146-1152.
35. Specka, U., Mayer, F. and Antranikian, G. (1991) Purification and properties of a thermoactive glucoamylase from Clostridium thermosaccharolyticum. Appl Environ Microbiol., **57**, 2317-2323.
36. Ganghofner, D., Kellermann, J., Staudenbauer, W. L. and Bronnenmeier, K. (1998) Purification and properties of an amylopullulanase, a glucoamylase, and an alphaglucosidase in the amylolytic enzyme system of Thermoanaerobacterium thermosaccharolyticum. Biosci Biotechnol Biochem., **62**, 302-308.
37. Takayanagi, T., Ajisaka, K., Takiguchi, Y., Shimahara, K. (1991) Isolation and characterization of thermostable chitinases from Bacillus licheniformis X-7u. Biochim Biophys Acta., **1078**, 404-410.
38. Bhushan, B. and Hoondal,G.S.(1998) Isolation, purification and properties of a thermostabl chitinase from an alkalophilic Bacillus sp. BG-11. Biotechnol Lett., **20**, 157-159.
39. Tsujibo, H., Endo, H., Miyamoto, K. and Inamori, Y. (1995) Expression in Escherichia coli of a gene encoding a thermostable chitinase from Streptomyces thermoviolaceus OPC-520. Biosci Biotechnol Biochem., **59**, 145-146.
40. Chien,A., Edgar, D.B. and Trela, J.M. (1976) Deoxyribonucleic acid polymerase from the extreme thermophile Thermus aquaticus. J Bacteriol., **127**, 1550-1557.
41. Kaledin, A.S., Sliusarenko, A.G., and Gorodetskii, S.I. (1980) Isolation and properties of DNA polymerase from extreme thermophylic bacteria Thermus aquaticus YT-1. Biokhimiya, **45**, 644-651.
42. Rudiger, A., Jorgensen, P.L. and Antranikian, G. (1995) Isolation and characterization of a heat-stable pullulanase from the hyperthermophilic archaeon Pyrococcus woesei after cloning and expression of its gene in Escherichia coli. Appl Environ Microbiol., **61**, 567-75.
43. Koch,R., Canganella, F. , Hippe, H. , and Jahnke, K. D. and Antranikian, G. (1997) Purification and propeties of a thermostable pullulanase from a newly isolated thermophilic anaerobic bacterium Fervidobacterium pennavorans Ven5. Appl Environ Microbiol., **63**, 1088-1094.
44. Linke, B., Rudiger, A., Wittenberg, G., Jorgensen, P.L. and Antranikian, G. (1992) Production of heat-stable pullulanase and a-glucosidase from the extreme thermophilic archaeon Pyrococcus woesi. DECHEMA Biotech Conf., **5**,161–3.
45. Costantino, H. R., Brown, S.H., and Kelly, R.M.(1990) Purification and characterization of an alpha-glucosidase from a hyperthermophilic archaebacterium, Pyrococcus furiosus, exhibiting a temperature optimum of 105 to 115 degrees C. J Bacteriol., **172**, 3654–60.
46. Rolfsmeier, M. and Blum, P. (1995) Purification and characterization of a maltase from the extremely thermophilic crenarchaeote Sulfolobus solfataricus. J Bacteriol., **177**,482–5.
47. Di, L.I., Morana, A., Ottombrino, A., Fusco, S., Rossi, M. and De, R.M. (1998) M. Enzymes from Sulfolobus shibatae for the production of trehalose and glucose from starch. Extremophiles., **2**, 409-16.
48. Ma, K., Robb, F.T. and Adams, M.W. (1994) Purification and characterization of NADP specific alcohol deydrogenase from the thermophilic archaeon Thermococcus hydrothermalis. Appl Environ Microbiol., **60**, 562-568.
49. Cannio, R., Fiorentino, G., Carpinelli, P., Rossi, M., and Bartolucci, S. (1996) Cloning and overexpression in Escherichia coli of the genes encoding NAD-dependent alcohol deydrogenase from two Sulfolobus species. J Bacteriol., **178**, 301-305.
50. Hei, D.J. and Clark, D. S. (1994) Pressure stabilization of proteins from extreme thermophiles. Appl. Environ. Microbiol., **60**, 932–999.
51. Hotta, Y., Ezaki, S., Atomi, H. and Imanaka, T. (2002) Extremely stable and versatile carboxylesterase from a hyperthermophilic Archaeon. Appl. Environ. Microbiol., **68**, 3925–3931.
52. Eckert, K. and Schneider, E. (2003) A thermoacidophilic endoglucanase (CelB) from Alicyclobacillus acidocaldarius displays high sequence similarity to arabinofuranosidases belonging tofamily 51 of glycoside hydrolases. Eur. J. Biochem., **270**, 3593–602.
53. Yumoto, I., Hirota1, K., Sogabe, Y., Nodasaka, Y., Yokota, Y. and Hoshino, T. (2003) Psychrobacter okhotskensis sp. nov., a lipase-producing facultative psychrophile isolated from the coast of the Okhotsk Sea. Int J. Syst. Evol. Microbiol., **53**, 1985–1989.
54. Maier, J., Kandelbauer,A., Erlacher,A. ,Paulo, A.C. and Gubitz, G. M. (2004) A new alkali-thermostable azoreductase from Bacillus sp. strain SF. Appl. Environ. Microbiol., **70**, 837–844.
55. Paar, A., Raninger, A., Sousa, F.D., Beurer,I.,Paulo, A.C. and Gubitz, G.M. (2003) Production of catalase-peroxidase and continuous degradation of hydrogen peroxide by an immobilized alkalothermophilic Bacillus sp. Food Technol. Biotechnol., **41**, 101–104.
56. Lipovova, P. K., Strnadb, H., Spiwoka, V. C., Kralova, B. and Russellc, N. J. (2003) The cloning, purification and characterization of a cold-active β-galactosidase from the psychrotolerant Antarctic bacterium Arthrobacter sp. C2-2. Enzyme Microb. Technol., **33**, 836–844.
57. Margesin, R., Sproer, C., Schumann, P. and Schinner, F. (2003) Pedobacter cryoconitis sp. nov., a facultative psychrophile from alpine glacier cryoconite. Int. J. Syst. Evol. Microbiol., **53**, 1291–1296.
58. Gomes, J. and Steiner, W. (1998) Production of a high activity of an extremely thermostable β-mannanase by the thermophilic eubacterium Rhodothermus marinus, Biotechnol. Lett., **20**, 729–733.
59. Taylor, I.N., Brown, R.C., Bycroft, M., King, G., Littlechild, J.A., Lloyd, M.C., Praquin, C., Toogood, H.S. and Taylor, S.J. (2004) Application of thermophilic enzymes in commercial biotransformation processes. Biochem. Soc. Trans., **32**, 290–292.
60. Birgisson, H., Delgado, O., Arroyo,L. G., Rajn,H.K. and Mattiasson, B.(2003) Cold-adapted yeasts as producers of cold-active polygalacturonases. Extremophiles., **7**, 185–193.
61. Thompson, V.S., Schaller, K.D. and Apel, W.A. (2003) Purification and characterization of a novel thermo-alkali-stable catalase from Thermus brockianus. Biotechnol Progr., **19**, 1292–1299.
62. Zhai, C., Cao, J. and Wang, Y., (2003) Cloning and expression of a pectate lyase gene from Bacillus alcalophillus NTT33. Enzyme Microb. Technol., **33**, 173–178.
63. Truong, .LV., Tuyen, H., Helmke, E., Binh, L.T. and Schweder, T. (2001) Cloning of two pectate lyase genes from the marine Antarctic bacterium Pseudoalteromonas haloplanktis strain ANT/505 and characterization of the enzymes. Extremophiles., **5**, 35-44.
64. Haukssona, J. B., Andressonb, O. S., and Asgeirsson, B. (2000) Heat-labile bacterial alkaline phosphatase from a marine Vibrio sp. Enzyme Microb Technol., **27**,66-73.
65. Svingor,A.,Kardos,J., Hajdu,I., Nemeth,A. and Zavodszky,P. (2001) A better enzyme to cope with cold. Comparative flexibility studies on psychrotrophic, mesophilic and thermophilic IPMDHS. J Biol Chem., **276**, 28121-28125.
66. Lonhiennea,T., Baisea,E., Fellera,G., Bouriotisb, G. and Gerdaya, C. (2001) Enzyme activity determination on macromolecular substrates by isothermal titration calorimetry: application to mesophilic and psychrophilic chitinases. Biochim Biophys Acta., **1545**, 349-356.
67. Li X, Yu HY (2011) Extracellular production of beta-amylase by a halophilic isolate, Halobacillus sp. LY9. J Ind Microbiol Biotechnol 38(11):1837-43.
